# Rare BK polyomavirus subtype III detected in hematopoietic cell transplant recipient

**DOI:** 10.1128/mra.00676-24

**Published:** 2024-09-30

**Authors:** Tiana A. Walder, Elizabeth A. Odegard, Heidi L. Meeds, Steven B. Kleiboeker, Assem Ziady, Anthony Sabulski, Sonata Jodele, Alix E. Seif, Stella M. Davies, Benjamin L. Laskin, Jason T. Blackard

**Affiliations:** 1 Division of Digestive Diseases, University of Cincinnati College of Medicine, Cincinnati, Ohio, USA; 2 Eurofins Viracor Laboratories, Lenexa, Kansas, USA; 3 Department of Pediatrics, University of Cincinnati College of Medicine, Cincinnati, Ohio, USA; 4 Division of Bone Marrow Transplantation and Immune Deficiency, Cincinnati Children’s Hospital Medical Center, Cincinnati, Ohio, USA; 5 Perelman School of Medicine, University of Pennsylvania, Philadelphia, Pennsylvania, USA; 6 Division of Oncology, Children’s Hospital of Philadelphia, Philadelphia, Pennsylvania, USA; 7 Division of Nephrology, Children’s Hospital of Philadelphia, Philadelphia, Pennsylvania, USA; Katholieke Universiteit Leuven, Leuven, Belgium

**Keywords:** BK polyomavirus, hematopoietic cell transplant, viral diversity, subtype

## Abstract

BK polyomavirus (BKPyV) is associated with disease in transplant patients. BKPyV genomic diversity is thought to contribute to functional differences in virulence factors. Here, we present the near full-length BKPyV genome sequence from a patient who underwent hematopoietic cell transplant and was infected with subtype III, a rare subtype.

## ANNOUNCEMENT

BK polyomavirus (BKPyV) is a member of the *Polyomaviridae* family and *Betapolyomavirus* genus. BKPyV is a non-enveloped, circular, double-stranded DNA virus with a global seroprevalence of up to 90% in adults (1). BKPyV primary infection most commonly occurs during childhood and is asymptomatic. The virus remains in a latent state until reactivation upon immunosuppression, typically following transplantation. Viral reactivation can lead to BKPyV-associated nephropathy and hemorrhagic cystitis in transplant recipients (1, 2). There are four subtypes of BKPyV. Subtype I is the most prevalent followed by subtype IV and then II and III, both of which are rarely found (3). BKPyV genomic diversity may contribute to functional differences in receptor binding and virulence, but its role is still not readily understood (4). Here, we present the near full-length BKPyV genome sequence extracted from the urine sample of a hematopoietic cell transplant (HCT) recipient infected with subtype III. Since this subtype is rarely observed or documented, enhancing the accessibility of data pertaining to less common subtypes is crucial.

The subject is a 9-year-old male born in Guatemala with a history of bone marrow failure secondary to Fanconi anemia who received an HCT from an unrelated donor. BKPyV viremia and viruria were detected in the first month after transplant with peaks of 600 copies/mL and 10 billion copies/mL, respectively. BKPyV viremia and viruria detection and quantification were performed at Viracor-Eurofins.

The urine sample at 1-month post-transplant was processed according to published methods (5). Briefly, viral DNA was extracted from 1 mL of urine. Rolling circle amplification was then performed on 1 µL of extracted DNA. The DNA was then linearized, and full-length PCR was performed using the primers BK1731F and BK1739R (6). The PCR product was run on a 1% agarose gel and extracted using the QIAquick Gel Extraction Kit.

Next-generation sequencing (NGS) was performed on PCR products. Library preparation was completed using the NEBNext Ultra II FS DNA Library Prep Kit and was sequenced on an Illumina NextSeq 2000 with the setting paired-end 2 × 61 bp. There were a total of 1,966,908 NGS reads. Quality control was analyzed using FastQC, and no reads were flagged as poor quality. All tools were run with default parameters. Reads were mapped to the reference genome V01108 (Dunlop) using UGENE 49.1 and the Bowtie2 method to generate a consensus sequence with an average depth of 16,606×, a length of 5,153 bp, and a GC content of 39.11%.

**Fig 1:**
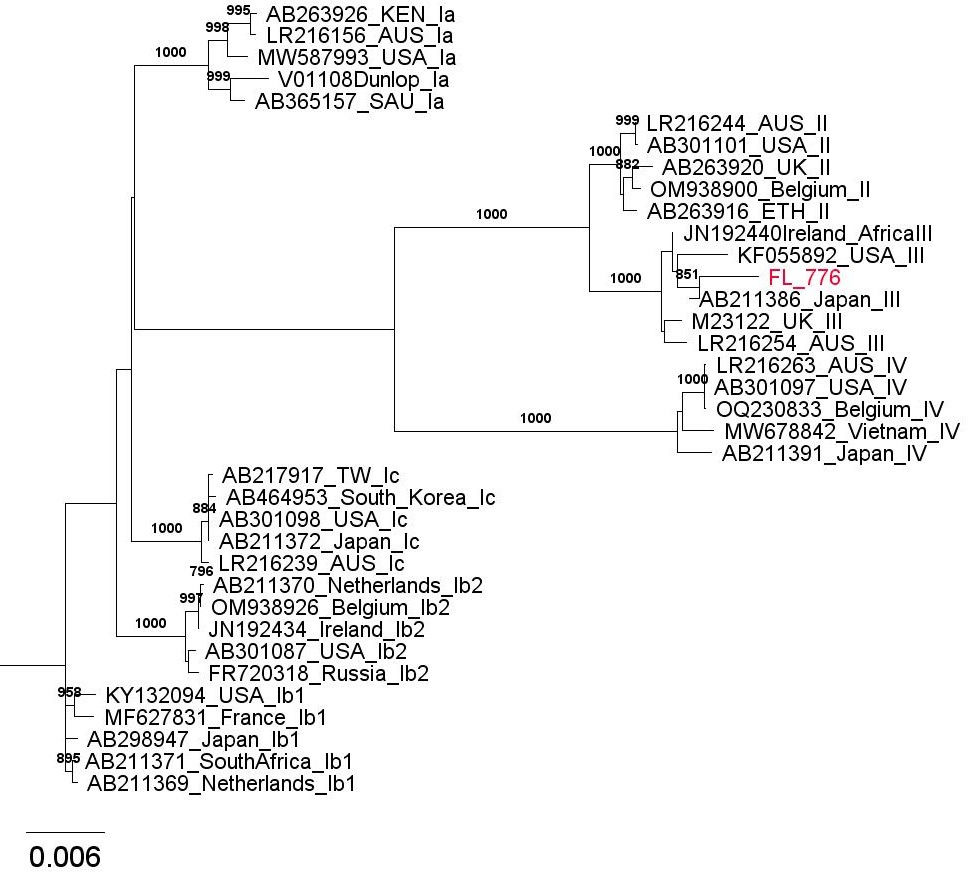
A multiple sequence alignment was performed in ClustalX 2.1 using pairwise alignment parameters, and a phylogenetic tree was generated using the neighbor-joining algorithm. Five representative reference sequences—from different countries of origin when available—were selected for each of the previously described BKPyV subtypes, including the four sub-genotypes of subtype I. References are labeled with their GenBank accession number, country, and BKPyV subtype. The sample sequence of interest is highlighted in red. Bootstrap values ≥ 700 have been included as branch labels.

A multiple sequence alignment was conducted in ClustalX 2.1 with the consensus sequence along with references of known BKPyV genotype extracted from GenBank. An unrooted phylogenetic tree was generated and visualized in FigTree. The patient sample clustered with subtype III references (Fig. 1). Pairwise genetic distances were calculated in Mega 11 using the Kimura 2-parameter model. There is a 99.62% nucleotide similarity between the sequence and the closest related reference sequence (AB211386) from Japan.

## Data Availability

Raw sequence data are available under BioProject PRJNA670723 with SRA accession number SRX24545791. The consensus BKPyV genome sequence is available under GenBank accession number PP798004.
